# Chelating Polymers for Targeted Decontamination of Actinides: Application of PEI-MP to Hydroxyapatite-Th(IV)

**DOI:** 10.3390/ijms23094732

**Published:** 2022-04-25

**Authors:** Jeanne Fèvre, Elena Leveille, Aurélie Jeanson, Sabine Santucci-Darmanin, Valérie Pierrefite-Carle, Georges F. Carle, Christophe Den Auwer, Christophe Di Giorgio

**Affiliations:** 1 CNRS, ICN, Université côte d’Azur, 06108 Nice, France; jeanne.fevre@univ-cotedazur.fr (J.F.); elena.leveille@univ-cotedazur.fr (E.L.); aurelie.jeanson@univ-cotedazur.fr (A.J.); christophe.denauwer@univ-cotedazur.fr (C.D.A.); 2 CEA, TIRO-MATOs, Université côte d’Azur, 06100 Nice, France; sabine.santucci@univ-cotedazur.fr (S.S.-D.); valerie.peirrefite@unice.fr (V.P.-C.); georges.carle@unice.fr (G.F.C.)

**Keywords:** actinides, decontamination, DTPA, chelating polymers, PEI-MP

## Abstract

In case of an incident in the nuclear industry or an act of war or terrorism, the dissemination of plutonium could contaminate the environment and, hence, humans. Human contamination mainly occurs via inhalation and/or wounding (and, less likely, ingestion). In such cases, plutonium, if soluble, reaches circulation, whereas the poorly soluble fraction (such as small colloids) is trapped in alveolar macrophages or remains at the site of wounding. Once in the blood, the plutonium is delivered to the liver and/or to the bone, particularly into its mineral part, mostly composed of hydroxyapatite. Countermeasures against plutonium exist and consist of intravenous injections or inhalation of diethylenetetraminepentaacetate salts. Their effectiveness is, however, mainly confined to the circulating soluble forms of plutonium. Furthermore, the short bioavailability of diethylenetetraminepentaacetate results in its rapid elimination. To overcome these limitations and to provide a complementary approach to this common therapy, we developed polymeric analogs to indirectly target the problematic retention sites. We present herein a first study regarding the decontamination abilities of polyethyleneimine methylcarboxylate (structural diethylenetetraminepentaacetate polymer analog) and polyethyleneimine methylphosphonate (phosphonate polymeric analog) directed against Th(IV), used here as a Pu(IV) surrogate, which was incorporated into hydroxyapatite used as a bone model. Our results suggest that polyethylenimine methylphosphonate could be a good candidate for powerful bone decontamination action.

## 1. Introduction

Actinide contamination may result from an incident in the nuclear industry, a malicious act targeting a nuclear power plant, the explosion of a dirty bomb, or a degraded situation in a nuclear-powered naval vessel such as an icebreaker or submarine. Although rare in the history of nuclear facilities, radioelement contamination is particularly harmful because these elements, all alpha emitters, present both chemical and radiological toxicity. Among these, plutonium (Pu) is particularly involved in both civil and military nuclear activity. It is highly toxic whatever its isotopy and remains an emblematic radioelement linked to the nuclear industry for the general public.

Despite the panic it may inspire, one should still consider that Pu is only/mainly toxic once it has entered the organism. Indeed, alpha emitters exhibit a limited power of penetration (e.g., about 5 cm in air and only about 30 µm, representing a few cell diameters, in living tissues); however, the deposited energy is enormous (alpha particles are on the order of MeV energy). Unfortunately, human exposure to Pu can be efficiently widespread through the inhalation of small particles kicked up by wind and dust after the accident has occurred.

Inhalation is, thus, the most likely entry route into the organism. The retention and the fate of the inhaled particles depend on their size and physicochemical form [1]. Bigger particles are either filtered by the upper respiratory region and swallowed or transferred into the throat by the lung clearance process (elimination of the mucus layer and particles through the natural motion of the bronchial cilia). In both cases, these particles are directed into the gastrointestinal tract and mainly excreted. On the contrary, the smaller particles (10 nm to 1 µm) are capable of reaching the lung alveoli [2], where they are sequestered by alveolar macrophages [3] and eventually transferred to lymph nodes or into lung tissues, representing long-term storage (for years), which contributes to lung cancers. The soluble forms (nitrates, citrates, and certain oxides) of inhaled Pu are absorbed more easily, pass into the bloodstream [4], and are redistributed throughout the body. About 90% of this absorbed Pu is then equally deposited in the liver and bones, where it contributes to the distribution of the dose over very long periods causing, in particular, bone cancer. In case of ingestion of Pu, subsequent entry into the bloodstream from the digestive tract is very low (<1%) [5]. Most of the ingested Pu is then eliminated in the feces [6].

As for the absorption of Pu through the skin, this represents a risk only for workers in highly contaminated areas and/or in the case of wounding (cut or blast injury). In that case, Pu follows the same paths as described earlier in the case of its transfer into the blood, whereby about 90% of the Pu absorbed is retained, mostly in the liver and bones.

Therefore, these three major compartments, lung, liver, and bones, constitute a real sanctuary for sequestered Pu.

The current recommended and approved treatment for contamination with transuranic radionuclides (e.g., plutonium, americium, and curium) is chelate calcium- and zinc-diethylenetriaminepentaacetic acid (Ca and Zn-DTPA), administered intravenously (i.v.) or by nebulizer. However, DTPA exhibits a narrow biodistribution [7,8] and, thus, is eliminated very quickly through urine [9]. Moreover, DTPA is only active on the soluble forms of Pu [10]. Of course, new chelating agents, some of which are promising, such as hydroxypyridonates (e.g., 3,4,3-LI(1,2-HOPO)) are still being developed [11]. However, as with DTPA chelation therapy, their action is mainly directed toward the soluble forms of Pu such as nitrates.

To address the limitations of DTPA, special formulations such as aerosolized Ca-DTPA for pulmonary administration have been tested [12,13]. Despite its effectiveness on soluble forms of Pu deposited in the lungs, it remained ineffective on insoluble oxides (PuO_2_) at the primary site of contamination. However, independently of the primary site of contamination or the DTPA treatment regimen (i.v. or by aerosolized Ca-DTPA), it reduced the systemic retention (skeleton and liver). Thus, there is still a need for a more effective treatment for the remaining Pu at the primary site of contamination.

Stealth liposomes encapsulating DTPA have also been tested [14] for their enhanced half-life in the blood and, hence, higher biodistribution. However, again, they showed improved efficiency when soluble forms such as ^238^Pu-phytate were injected. As for the bones, to the best of our knowledge, only one team [15] reported a real in vivo decontamination, of uranium only, using a 3,2-hydroxypyridinone-based compound. Indeed, the lower observed actinide level in the liver and/or bones highlighted in most studies should be rather attributed to the chelation effect (subsequent to the i.v. administration) of the decontaminating agent rather than to actinide extravasation (e.g., real decontamination) from the liver and/or bones. In other words, once the Pu has been incorporated into a retention compartment, it is virtually inextricable.

In spite of its limitations (effectiveness mainly confined to circulating soluble forms of Pu), i.v. DTPA remains the most efficient treatment and constitutes a solid basis for comparison. It should be noticed that DTPA decontamination therapy is also quite well tolerated since, according to the National Council on Radiation Protection and Measurements, NCRP (Report No. 166), Bethesda, MD, 2011, the recommended dose (i.v. or nebulized inhalation) represents 1 g (in one shot) of chelate/day, while, in case of multiple treatments, total amounts as high as 500 g of DTPA could be administrated within several years. However, at the present time, no treatment fully meets all the required specifications. More specifically, no agent is currently capable, in addition to its action in the bloodstream, of (i) specifically targeting the three major biological Pu retention compartments, and (ii) extracting it from there.

Our challenge was then to propose a simple, effective, and affordable complementary method to DTPA therapy based on a polymeric platform. We postulated that a chelating polymer would indirectly target the main organs (liver and lungs) or the bones (provided an affinity for bone fixation sites would be implemented in that case). This strategy could, therefore, be entirely complementary to Ca-/Zn-DTPA therapy which, as previously seen, is the only one currently in use despite being mainly effective for the circulating (e.g., soluble) forms of Pu.

**Figure 1:**
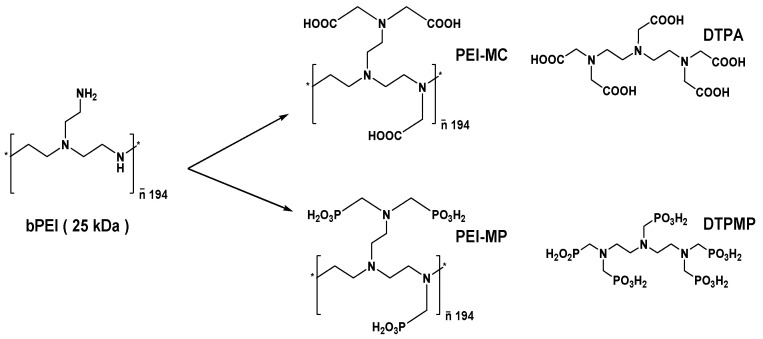
Chemical structures of the chelating polymers carrying carboxylic or phosphonic functions and their reference molecular analogs.

During the past years, our group has been developing a macromolecular approach based on a polyethyleneimine backbone for the decontamination of actinides [16,17,18,19]. This can provide, after adequate functionalization, polymeric analogs of DTPA, PEI-MC (polyethyleneimine methylcarboxylate, true polymeric analog) and PEI-MP (polyethyleneimine methylphosphonate, phosphonate analog) from a commercially available 25 kDa branched PEI (polyethyleneimine, see Figure 1). One fully functionalized polymer is most likely represented by a wide polydisperse population with a mass fraction ranging from ~5–150 kDa.

The syntheses of these chelating PEIs can be carried out in a single step, with very high yields, and are very cheap. Purification via ultrafiltration is straightforward. Too many chelating agents developed so far require a greater number of tedious synthesis and purification steps. Furthermore, as these compounds are polyelectrolytes salts, they readily dissolve in water, making them very easy to use.

We have clearly demonstrated the ability of PEI-based polymers to sequester both U(VI) (uranyl) and actinides(IV) (Pu and Th as a chemical surrogate of Pu). Indeed, under (pseudo)physiological conditions, the two polymers show an EC_50_ (50% effective concentration) comparable to DTPA taken as a reference. Complexation to Pu(IV) was also demonstrated by EXAFS spectroscopy with both polymers, PEI-MC and PEI-MP [18,19].

We present now, in this report, a first study on the decontamination of Th(IV) from a hydroxyapatite (HAp) matrix in which Th(IV) has been used as a chemical surrogate of Pu(IV) (see Section 2.4). Dose–response and kinetic profiles of decontamination associated with this model are provided. Furthermore, viability experiments realized in bones constitutive cells (osteoblasts and osteoclasts) are also reported for PEI-MC and PEI-MP compared to DTPA, taken as the gold standard.

## 2. Results and Discussion

### 2.1. PEI-MP as a Bone Seeker

The development of radiopharmaceuticals with specific bone-targeting abilities, such as ligand–radionuclide conjugates, has been carried out extensively considering metastatic bone cancer. In that case, the ligand should not only prevent radionuclide dissociation but also exhibit a strong affinity for the principal mineral phase of bone, hydroxyapatite (HAp). Indeed, HAp which is a calcium phosphate, Ca_5_(PO_4_)_3_(OH), constitutes 70% of the bone weight. HAp, then, undoubtedly constitutes a robust structural and chemical model for studying the actinide decontamination process. Phosphonates show a particular affinity for Ca^2+^. As a consequence, lower and higher (di)phosphonate analogs of DTPA have been considered as ligands with bone-seeking properties for diverse radionuclides (^153^Sm, ^117m^Sn, ^99m^Tc). For example, the beta emitter ^153^Sm complexed with ethylenediaminetetramethylene phosphonate (Quadramet^®^) found a clinical use with osteoblastic skeletal metastases [20]. To improve radionuclide import, Zeevaart et al. showed that PEI-MP, loaded to some extent with radionuclides (^99m^Tc, ^153^Sm), could target the bone after i.v. injection in dogs [21]. Dormehl et al. demonstrated, in primates, that t_1/2_ and percentage of uptake of the ^99m^Tc–PEI-MP complexes, in diverse compartments, could be modulated by manipulating the mass fraction of the polymer. The same study also reported on the excretion of these complexes through the kidneys related to diverse mass fractions (kDa) of the polymer [22]. From these studies, they concluded that the fraction size 10–30 kDa seems to be the most suitable for radioisotopic therapy, with a lower uptake and shorter t_1/2_ in liver and kidneys while having the highest bone uptake.

In the case of actinide contamination, particularly in the case of bone invasion by Pu(IV), we envisioned a similar approach with the use of chelating polymers as potential candidates. PEI-MP in particular should exhibit bone-seeking abilities. After adsorbing onto bone (i.e., HAp), it could behave as a decontaminant and extract the actinide(IV) provided the thermodynamic and kinetic profiles (adsorption/desorption of ligand and complexes) are compatible/tunable with the targeted biological utilization. As the liver or even the kidneys (in the case of contamination by U) could also be taken as relevant targets, our chelating polymers were not fractionated except for the smallest sizes (<5 kDa), which were eliminated via ultrafiltration (see Section 3).

### 2.2. Toxicity of PEI Chelates toward Bone Constitutive Cells

**Figure 2:**
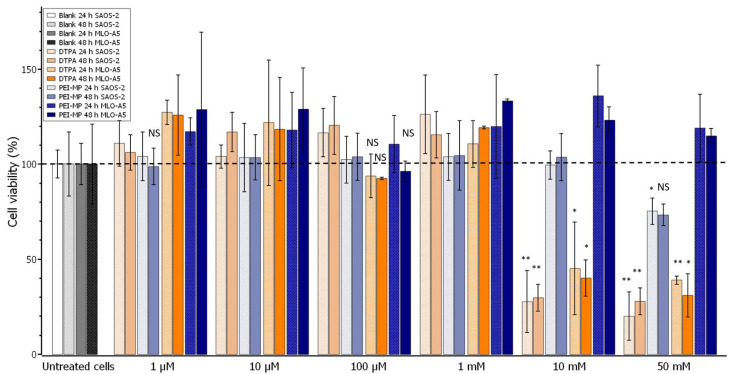
Graph representing the mean percentage of cell viability for MLO-A5 or SAOS-2 cells treated with increasing concentrations of chelates for 24 or 48 h. Error bars represent the SD. The mean and the SD were calculated from independent experiments (*n* = 3). * *p* < 0.05; ** *p* < 0.005, NS (not significant), according to *t*-test results (equal means) of DTPA or PEI-MP when inferior to their respective blank (untreated MLO-A5 or SAOS-2 cells at the corresponding time).

Bones undergo a constant cycle of construction and resorption, which must remain in equilibrium. Many types of cells and factors are involved in this process. Osteocytes and osteoblasts from mesenchymal stem cells are in charge of matrix formation and mineralization, whereas osteoclasts ensure the resorption process. The whole process constitutes the bone homeostasis. The potential decontamination of actinides through this chelation therapy should ideally be highly tolerated by the constitutive cells. Accordingly, viability was assayed onto SAOS-2 osteoblastic cells and MLO-A5 cells (osteocytes). The results are presented in Figure 2.

Interestingly, PEI-MP did not show any toxicity regardless of cell type or dose utilized, except at the highest dose, 50 mM, on SAOS-2 osteoblasts, where we observed a very slight effect (80%), despite the result at 48 h not showing a significant statistical difference with the untreated cells. On the contrary, DTPA was toxic from 10 mM toward both cell lines. This result, together with the evidence of PEI-MP excretion provided by Dormehl et al. [22], is very encouraging given that, when used in vivo, PEI-MP does not show any serious deleterious effects.

### 2.3. Affinity toward HAp

To assess the targeting abilities of the chelates for the bones, their affinity toward a hydroxyapatite powder was measured by thermogravimetric analyses. We, thus, monitored the mass percentage degradation of the organic matter, in the 250–500 °C range, due to PEI-MP, PEI-MC, and DTPA (as a control) after being immobilized on HAp.

**Figure 3:**
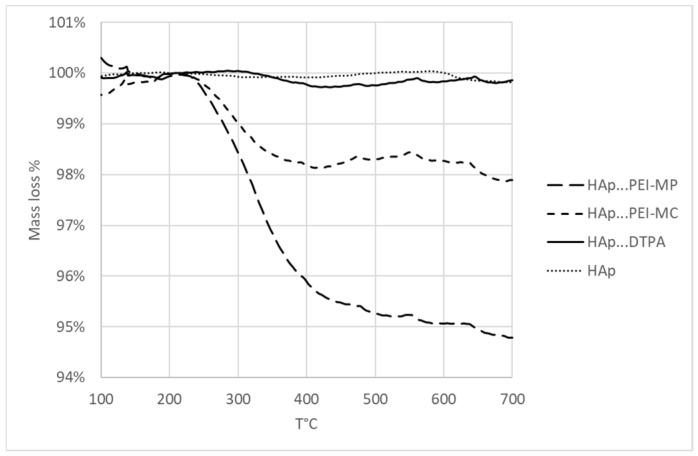
Thermograms of HAp, HAp…PEI-MP, HAp…PEI-MC, and HAp…DTPA in the 100–700 °C range showing the percentage of mass loss due to the organic part (chelates).

Figure 3 shows the thermogravimetric analysis curves for each chelating molecule. For reading convenience, each thermogram was normalized so that 100% fit the initial mass loss plateau (around 200 °C). Keeping the initial ratio of chelating monomer to HAp constant, the HAp mass loss exhibited the expected trend with increasing loss from DTPA to its homolog PEI-MC and then to the phosphonate analog PEI-MP. Maximum adsorption onto HAp was observed with PEI-MP with a mass loss of about 4.7%, whereas the degradation due to PEI MC corresponded to about 1.8%. As for the DTPA, adsorption was negligible (0.3%). This experiment confirmed that the chelating polymers showed a higher affinity toward the target HAp. One can calculate that PEI-MC and PEI-MP exhibited sixfold and 15-fold higher affinity, respectively, for the HAp matrix as a bone model than DTPA. An affinity of 0.042 µmol of PEI-MP/µmol of HAp could be calculated.

### 2.4. Contamination of HAp

In case of contamination, postmortem radiometry showed that 90% of plutonium activity in extrapulmonary sites was deposited in the liver and skeleton. Additionally, the fraction of plutonium deposited in the skeleton increased with time [23]. Decontamination of Pu(IV) from the bones remains of particular interest especially, for workers who have suffered from a wound while handling irradiated material.

To facilitate both handling and radioprotection, the experiments were carried out with Th(IV) as a chemical surrogate of Pu(IV), which is the major form of Pu in biological media. Th is indeed easier to manipulate (6 log lower specific activity for ^232^Th compared to ^239^Pu) while exhibiting the same +IV oxidation state with a comparable ionic radius [24,25]. This is very important since chelation and desorption of complexes from the HAp matrix strongly depend on these parameters. Nonetheless, this approach must be considered with some care, and test experiments with Pu will possibly be performed after full optimization with Th.

HAp was contaminated with Th(IV) through a reverse ionic exchange process. The Th(IV) source was provided by thorium carbonate, generated in situ from its nitrate form, in sodium carbonate excess, under the chemical form of the pentacarbonate complex [Th(CO_3_)_5_]^6−^. A contact time of 48 h with HAp was set to ensure equilibrium was reached. After purification, the extent of Th^4+^ replacement at Ca^2+^ was measured by ICP-MS analyses. Thus, 35 ± 1.2 µg (*n* = 3 on six independent HAp-Th samples) of elementary Th (0.7% of initial Th) was found to be incorporated in 5 mg of HAp. Considering that 5 mg of HAp contained 49.75 µmol of Ca, this corresponds to a molar ratio Th/Ca of almost 0.003. Furthermore, the speciation of the Th(IV) cations into the HAp was investigated via EXAFS spectroscopy to make sure all the Th was fully incorporated (chemisorbed) into the matrix and not simply physisorbed as a carbonate complex. This spectrum constitutes a reference to be compared with those from the chelate–Th complexes, DTPA–Th, PEI-MC–Th, and PEI-MP–Th, after the decontamination process was carried out.

**Figure 4:**
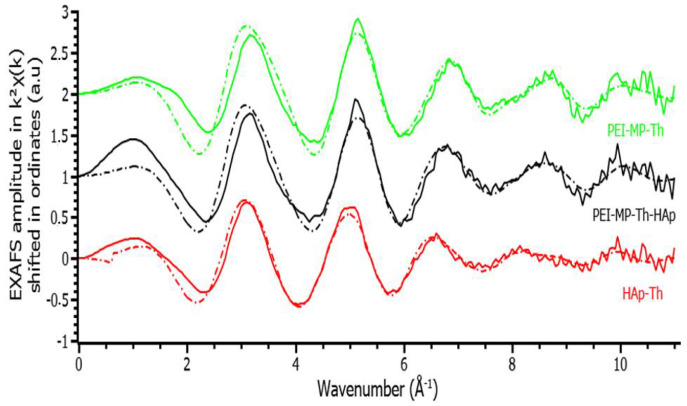
Th L_III_ edge experimental EXAFS spectra of PEI-MP–Th (green), PEI-MP–Th–HAp (black), and HAp–Th (red). Fits are shown in dotted line.

The EXAFS spectrum of HAp–Th is presented in Figure 4, and the corresponding best-fit parameters are displayed in Table 1.

The fitting procedure included the contributions of oxygen and phosphorus atoms from the phosphate groups, showing that the Th atoms were incorporated in the phosphate matrix. The average distance of the oxygen contributions of the first coordination sphere (2.45 Å) was close to the average distance in the phosphate diphosphate Th_4_(PO_4_)_4_P_2_O_7_ (2.44 Å) [26]. The short phosphorus distance (3.21 Å) is in agreement with bidentate phosphate groups, while the longer phosphorus contribution (3.82 Å) would correspond to monodentate phosphate groups as in the phosphate diphosphate phase. Adding a Th–Th contribution did not significantly improve the fit, meaning that the Th atoms were dispersed in the apatite phase and did not form clusters.

Surprisingly, a similarly good fit could be obtained by replacing one of the phosphorus contributions with C and O (distal) contributions of a carbonate anion (R_factor_ = 2.6%). Thus, the EXAFS spectrum did not allow us to conclude on the presence or not of carbonate anions in the Th coordination sphere within the apatite matrix. Indeed human bone may contain up to 8 wt.% carbonate ions that occupy phosphate and hydroxide positions in the HAp lattice [27]. Note, however, that the spectrum could not be adjusted with carbonate contributions only (like in the pentacarbonato complex [28]), which confirmed that thorium in HAp was not in the form of pure carbonate complexes. In conclusion, our sample preparation incorporated Th in the HAp matrix in the form of a Th phosphate disordered phase with the possible occurrence of carbonate anion(s) in its coordination sphere.

### 2.5. Thermodynamics of HAp–Th Chelate Systems: Dose–Response Curves

The capacity of the two chelating polymers to extract Th from the contaminated HAp was measured, at day 8, in TBS (50 mM Tris, 150 mM NaCl, pH 7.4) at different monomer concentrations and compared to DTPA as a reference by titrating the elementary Th (ICP-MS) present in the filtrate and, hence, complexed to the chelating agents.

**Figure 5:**
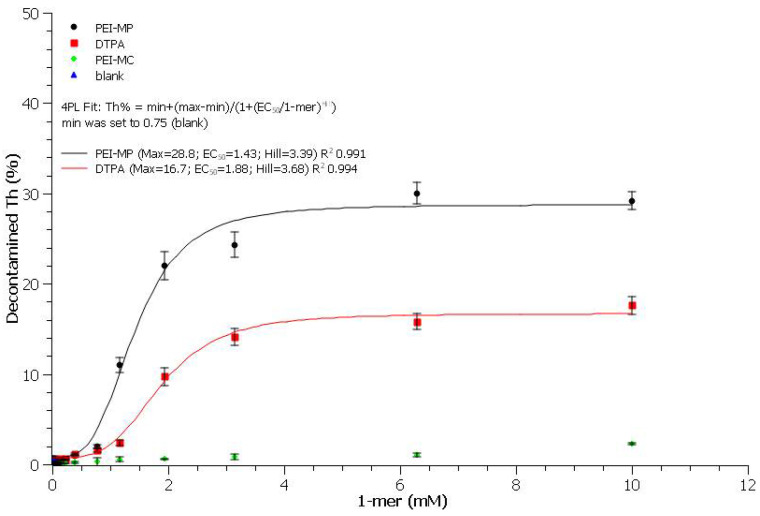
Dose–response curves of HAp–Th (5 mg, 0.7% Th) versus monomer chelate in 1.5 mL of TBS at day 8.

The uptake curves of Th(IV) from contaminated HAp–Th with PEI-MP, PEI-MC, and DTPA are shown in Figure 5 and adjusted using a four-parameter logistic equation: response = min + (max − min)/(1 + (EC_50_/dose)hill), where response is the Th% decontaminated, dose is the monomer concentration, min is the response in the absence of chelate (blank), max is the plateau, EC_50_ is the efficient concentration required to produce 50% response, and hill is the slope at the EC_50_. Very importantly, for each experiment, the Th content remaining bound to HAp was also determined to ensure 100% recovery (see Supplementary Materials).

First of all, despite a better affinity for HAp (sixfold when compared to DTPA; see thermogravimetric results), PEI-MC showed unexpected outcomes. Indeed, the maximum Th content, 2.3%, recovered from the filtrate (green diamonds in Figure 5) was quite low, as with the blank (0.75%, at 0 mM). This could be due either to much more unfavorable desorption kinetics of the PEI-MC/Th complexes from the HAp or to a precipitation of these complexes. Secondly, despite similar values of EC_50_ and hill factors (i.e., the slope at the EC_50_ describing the transition rate from the concave to convex part of the sigmoid), the efficiency (maximum value at the plateau) strongly differed between the PEI-MP and the reference. Both of them undoubtedly showed decontamination abilities, but the phosphonate polymer was almost twice as effective: 29% versus 17% for DTPA (test for equal means provided a probability *p* (same mean) < 0.005). This result is undoubtedly significant. Indeed, DTPA is unlikely to reach the sequestering actinides site because of its low bioavailability, unless it is injected locally in the vicinity of the contaminated bone. However, even in this particular case, its activity would be limited due to its low affinity for the mineral part of the bone matrix, i.e., HAp. On the contrary, the thermodynamics of PEI-MP is more favorable, with higher affinity toward HAp and twofold better efficiency at extracting Th(IV). Furthermore, biodistribution, t_1/2_, affinity, and uptake are all compatible with the envisioned objective [22].

The minimal monomer concentration needed for maximum decontamination under experimental conditions was determined and set at 6.3 mM, just after reaching the plateau and below the toxicity threshold of DTPA. This concentration, corresponding to a monomer/Th molar ratio of ~60, was used for studying the kinetics of decontamination with the chelates (see here after).

### 2.6. EXAFS of PEI-MP–Th and PEI-MP after Contact with HAp–Th

The fit of the EXAFS spectrum of PEI-MP–Th is shown in Figure 4, and best-fit parameters are provided in Table 1. Distances are characteristic of monodentate phosphonate coordination from a Th–phosphopeptide complex (2.35 Å for the Th–O(P) distance) that has already been described in a previous study [17]. It is quite remarkable that, using different sample preparations (present study from Th carbonate, previous study from Th NTA), very similar parameters were obtained. The presence of additional Cl anions in the second sphere is surprising but arose from the high chlorine content of the physiological medium (150 mM). This was also observed in Lahrouch et al. [17].

The EXAFS spectrum of HAp–Th in contact with PEI-MP was compared with the EXAFS spectra of HAp–Th and PEI-MP–Th (see Figure 4). Best-fit parameters were very similar to those obtained for PEI-MP–Th, meaning that Th incorporated into the HAp matrix changed speciation in the presence of PEI-MP. The resulting coordination sphere was similar to the environment of Th when complexed directly with PEI-MP alone and also similar to the previous preparation from Th-NTA. This confirmed the ability of PEI-MP to extract Th from the HAp matrix.

### 2.7. Kinetics of HAp–Th Chelate Systems

**Figure 6:**
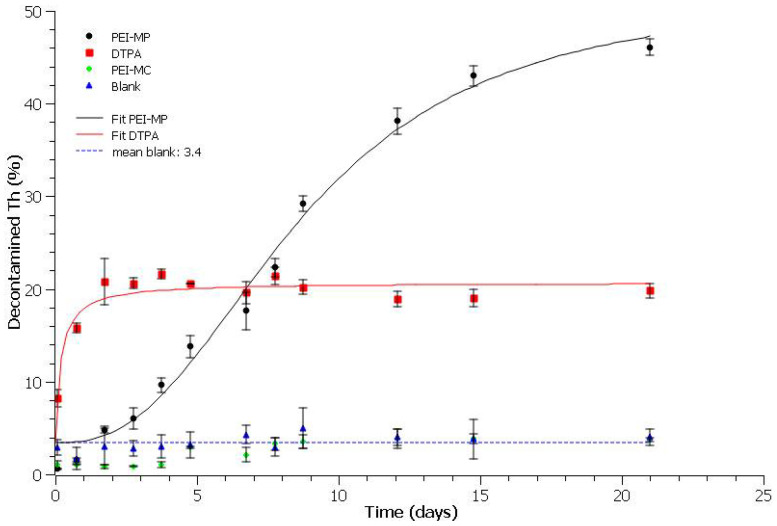
Decontamination kinetics of HAp–Th (5 mg, 0.7% Th) in the presence of 6.3 mM chelate in 1.5 mL of TBS over 21 days.

We then investigated kinetics, at 6.3 mM (minimal experimental monomer concentration to reach the highest Th extraction with both DTPA and PEI-MP) to see whether or not it would be possible to enhance the maximum efficiency obtained at day 8. The results are presented in Figure 6. Again, complementary Th content was checked to ensure 100% recovery (see Supplementary Materials). As for thermodynamics, curves were fitted using the same equation in Section 2.5 with the “min” parameter set to 3.4 (mean blank value across the whole time period).

First of all, decontamination due to Th solubilization and/or release from HAp only was marginal (3.4% ± 1.3%), as seen with blank samples across the time period. This mean value could be attributed to the phosphate ions released from HAp at equilibrium in TBS. Secondly, the extraction of Th content with PEI-MC remained very inefficient with values of the same order of magnitude as the blanks. Most importantly, here again, huge behavior differences could be drawn between DTPA and PEI-MP. DTPA decontamination kinetics were very fast, with the highest value by day 2, but reached a plateau at 20% that could not be exceeded whatever the incubation time. It should be noted that this maximum efficiency value could not be enhanced even with a higher concentration of the chelate, as seen before with the dose–response curve. On the contrary, the kinetics with PEI-MP were much slower. Thus, the effectiveness of DTPA was matched by days 6–7. However, contrary to the DTPA trend, the Th decontamination increased across the used time period of 21 days. Strikingly, the maximum efficiency reached a top value of 46% at day 21 (*p* (same mean as DTPA) < 0.005). Furthermore, it should be noted that Th extraction at day 8, 29%, remained consistent with the value found during the dose–response experiment (30%). Additionally, when the filtrate was replaced at day 12 (35.8% Th extraction) with a fresh amount of 6.3 mM monomer PEI-MP, the cumulative Th decontamination reached 64.9% at day 21 (see Table 2). This indicates that additional treatment with PEI-MP over time could allow for almost a full recovery of all the mobilizable Th content.

Most importantly, these results also indicated that, unlike DTPA, the desorption of PEI-MP/Th complexes from the contaminated HAp was a slow time-dependent process that, once the effective dose was reached, continuously dragged the Th out of the HAp matrix. It should be noted that we set up here a particularly unfavorable situation where the HAp was highly contaminated (0.7% Th). Despite these conditions, PEI-MP showed an interesting ability to extract Th(IV) even though the kinetics were slow. In vivo, this could eventually be compensated for/modulated by its higher affinity for the HAp, by adjusting the polymer mass fraction and, hence, the desorption equilibrium from HAp and/or by using it in combination with the DTPA.

## 3. Materials and Methods

### 3.1. Reagents

Bromoacetic acid, sodium carbonate, acid chloride (37%), phosphorous acid H_3_PO_4_, branched polyethyleneimine (bPEI, 25 kDa MW), formaldehyde (37% solution), and hydroxyapatite nanopowder (<200 nm particle size (BET), ≥97%, synthetic) were purchased from Sigma-Aldrich, Saint-Louis, Mo, USA. This hydroxyapatite (HAp in the text) was provided with the following elemental analysis: Ca, 39.89%; H, 0.20%; O, 41.41%; P, 18.50%, corresponding to a formula of Ca_5_(OH)(PO_4_)_3_, and it was kept under inert atmosphere. Nitric acid (67–70%, Plasmapure plus degree) and ICP-MS standard solution were purchased from SCP Science, Villebon sur Yvette, France and Honeywell Fluka, Guyancourt, France. A Th(IV) stock solution was prepared from thorium nitrate solution (5.8 mg Th(NO_3_)_4_.5H_2_O in 1 mL of HNO_3_ 0.1 M, (Th(IV)) = 0.01 M). Ultrafiltration was carried out with a stirred ultrafiltration cell (Millipore, 76 mm) equipped with ultrafiltration membrane disc filters Omega™ membrane, OM005076, 5 kDa MWCO (Pall corporation, port Washington, NY, USA) for the polymer purification. Ionic chromatography was performed with a Metrohm, Villebon sur Yvette, France 761 compact apparatus, equipped with a Metrosep Anion Dual 1 (3 × 150 mm) column, using 2.4 mmol/L NaHCO_3_/2.5 mmol/L Na_2_CO_3_ + 2% acetone (conductivity after chemical suppression approximately 16 µS/cm) as eluent for the determination of chloride content. Na^+^ counterions and phosphorus content were determined from ICP-AAS optima 8000 (Perkin Elmer, Villebon sur Yvette, France) with, respectively, Na standard solution and ICP P standard solution (Honeywell Fluka, Guyancourt, France) (see specific conditions below). Quantification of the thorium content was carried out by resorting to external calibration with standards of Th in HNO_3_ 1% prepared from ICP-MS Th standard solutions plasmaCAL. (SCP Science, Villebon sur Yvette, France). All ICP-MS experiments were performed with ELAN 9000 ((Perkin Elmer, Villebon sur Yvette, France).

### 3.2. Synthesis of PEI Chelates

#### 3.2.1. Synthesis of PEI-MC

Branched PEI 25 kDa (10 g, 77.52 mmol of monomeric units, C_6_H_15_N_3_) was dissolved in 500 mL of sodium carbonate solution (0.1 M). Then, bromoacetic acid (53.9 g, 387.6 mmol, 5 eq) diluted in water was added dropwise under constant stirring. The reaction mixture was stirred at room temperature overnight. The resulting solution was acidified with HCl to pH ≈ 7, and then purified by ultrafiltration (5 kDa MWCO) with a three-step sequence procedure. First, the neutral reaction mixture coming from the functionalization step was passed through the 5 kDa MWCO membrane (under adequate pressure); the resulting residue was then rinsed thoroughly with a saturated solution of NaCl (250 mL) and then with ultrapure water (250 mL × 2). Finally, the product was freeze-dried and conserved under inert (argon) to prevent moisture addition. This yielded 26 g of the water soluble polyethyleneimine methylcarboxylate sodium salt. Microanalysis revealed a C/N mass ratio of 3.43 (C/N molar ratio of 4) indicating that a full level of methylene carboxylation was achieved. Furthermore, the counterion amount, sodium for carboxylate and eventually chloride for tertiary amine, was determined with ICP-AAS and ionic chromatography, respectively (see Supplementary Materials). The following molecular formula per monomer was derived: C_12_H_19_N_3_O_6_Na_2_ (MW 347.28 g/mol), suggesting that the dried polymer, as a sodium salt, dissociated into polyampholyte.

#### 3.2.2. Synthesis of PEI-MP

PEI-MP was synthesized as described elsewhere [29]. Basically, phosphorous acid H_3_PO_4_ (19.1 g) was dissolved in concentrated HCl solution (50 mL) and heated at 80 °C. Then, formaldehyde 37% (37.8 mL) was added dropwise. Branched 25 kDa PEI (10.0 g) was dissolved in water (48 mL), and this solution was added dropwise to the reaction mixture. The reaction mixture was stirred at 90 °C for 2 h and then cooled slowly overnight. The product was separated as a viscous oil. After decanting, this viscous oil was washed with water to form a doughy substance. This decanting/washing procedure was repeated twice. The resulting oily residue was basified with Na_2_CO_3_ to pH ≈ 5, concentrated under vacuum, and then purified by ultrafiltration (5 kDa MWCO) as above, before being freeze-dried and conserved under inert (argon) to prevent moisture addition. This yielded 7.8 g of the water-soluble polyethyleneimine methylphosphonate sodium salt). Microanalysis revealed a C/N mass ratio of 2.57 (C/N molar ratio of 3) indicating that a full level of methylene phosphonylation was achieved. As for the PEI-MC, counterions were determined with ICP-AAS and ionic chromatography (see Supplementary Materials). The following molecular formula per monomer was derived: C_9_H_21.5_N_3_P_3_O_9_Na_3.5_ (MW 488.16 g/mol), suggesting that the dried polymer, as a sodium salt, dissociated into polyampholyte.

### 3.3. Toxicity of PEI Chelates toward Bone Constitutive Cells

The SAOS-2 cell line was purchased from the American Type Culture Collection. Briefly, SAOS-2 cells were maintained in McCoy’s 5A medium without phenol red (HyClone, Thermo Fisher Scientific, Waltham, MA, USA) supplemented with 15% heat-inactivated fetal bovine serum (Biowest, Nuaille, France) and antibiotics (100 IU/mL penicillin, 100 µg/mL streptomycin, Sigma-Aldrich, Saint-Louis, MO, USA). Prior to assessment of toxicity, SAOS-2 cells were plated in 96-well plates (12,000 cells/well).

The MLO-A5 (murine-like osteocytes) cell line obtained from Linda Bonewald lab [30] was maintained at 37 °C, 5% CO_2_ using rat tail collagen I-coated (Sigma-Aldrich, Saint-Louis, MO, USA) wells, in α-MEM with nucleotides and Ultraglutamine (BE02-002F, Lonza, Basel, Switzerland) supplemented with 1% penicillin/streptomycin (P/S, Sigma-Aldrich, Saint-Louis, MO, USA), 5% heat-inactivated fetal bovine serum (FBS: Hyclone SH30071.03 GE Healthcare, Chicago, IL, USA) and 5% heat-inactivated calf serum (CS: Hyclone SH30072.03, GE-Healthcare, Chicago, IL, USA).

For the assessment of toxicity, the medium was replaced by complete culture medium supplemented with DTPA, PEI-MP, or PEI-MC (0.1 µM, 1 µM, 10 µM, 100 µM, 1 mM, 10 mM, 50 mM, and 100 mM). Cells were further incubated for 1 h and washed three times with PBS buffer to remove the noninternalized polymers or DTPA. Next, 200 µL of culture medium was added, and cells were incubated for 24 or 48 h. At the end of the incubation period, cytotoxicity was assessed using the MTT assay. Briefly, culture medium was removed and replaced by 100 µL of EMEM containing MTT. After 1 h at 37 °C, EMEM containing MTT was removed and replaced by 150 µL of DMSO. After 15 min, absorbance was measured at 570 nm. The mean absorbance of nonexposed cells was taken as the reference value. The percentage of cell viability was calculated on the basis of the ratio between the absorbance of each sample compared to the average absorbance of the untreated cells. Results were expressed as the percentage mean (±SD) from two independent experiments performed in triplicate.

### 3.4. Determination of the Affinity of the Chelating Agents toward HAp by Thermogravimetric Analysis

HAp powder (10 mg) was dispersed in a 1.5 mL solution of DTPA, PEI-MC or PEI-MC (10 mM, based on monomer concentration), 1.5 µmol monomer/mg, and stirred overnight. Samples were then centrifuged at 15,000 rpm during 15 min and washed (three times), to remove unbound DTPA, PEI-MC, or PEI-MP. The resulting HAp–chelate powder was freeze-dried to eliminate the water excess and kept under inert atmosphere. Thermogravimetric analyses (TGA) were performed on a Mettler Toledo, Columbus, OH, USA, TGA 851e using STAR© software (version 13.00) for data analysis. Freeze-dried samples (~10 mg) were placed in 70 µL alumina pans and heated at 10 °C·min^−1^ from 25 to 800 °C under N_2_ flow (50 mL·min^−1^). Calculation of the mass loss percentage, in the 200–400 °C range, allowed directly determining the affinity of each chelate toward HAp their comparison.

### 3.5. Contamination of HAp

To avoid hydrolysis at physiological pH, Th(NO_3_)_4_, was converted into Th(CO_3_)_4_. Briefly, a Th(CO_3_)_4_ (1.33 mM) solution was prepared by adding 400 µL of Th(NO_3_)_4_ (0.1M) in 29.6 mL of Na_2_CO_3_ (0,1M). The resulting Th(IV) solution, 30 mL (1.33 mM), was then incorporated into the hydroxyapatite (HAp) (500 mg). This suspension was stirred for 48 h. The contaminated HAp–Th powder was submitted to three cycles of centrifugation/washing steps with ultrapure water until no Th could be detected (ICP-MS) into the last filtrate (see Supplementary Materials). HAp–Th powder was then freeze-dried to yield 480 mg of a white powder. The incorporation level of Th(IV) into the HAp was ensured by quantifying the elementary Th via ICP-MS from different aliquots of the HAp–Th powder. It should be noted that the incorporation level of Th(IV) into the HAp matrix could be very precisely controlled using this procedure. This procedure was independently repeated onto different HAp samples, and contamination rates were found to be highly repeatable. Overall, the total Th content into the contaminated HAp samples was 0.7%.

### 3.6. Efficiency of Th Extraction: Dose–Response of HAp–Th Subjected to the Chelates

First, 5 mg of HAp–Th powder (0.7% Th content as determined by ICP-MS) were added to 1.5 mL of chelates (PEI-MC, PEI-MP, or DTPA), at different concentrations, in TBS buffer (50 mM Tris, 150 mM NaCl, pH 7.4) and mixed (orbitally) for 7 days. The corresponding chelate monomer molar concentrations were equal to 0 (blank), 0.010, 0.015, 0.039, 0.050, 0.077, 0.15, 0.23, 0.39, 0.77, 1.16, 1.93, 3.14, 6.29, and 10.0. Each of these samples was prepared in triplicate. After 8 days, a purification step (15,000 rpm, 15 min) was carried out. Then, 500 µL of supernatant was recovered from two centrifugations (250 µL 2×) for each sample and digested in 5 mL of 67–70% HNO_3_ (Plasmapure plus degree, SCP Science, Villebon sur Yvette, France) at 120 °C during 2 h. The digested samples were then evaporated to dryness at 90 °C using a heating block. Finally, 5 mL of 1.5% HNO_3_ was added to the tubes. Each sample was analyzed by ICP-MS (Perkin Elmer ELAN 9000). Operation conditions were daily optimized using a tuning solution. Determination of the Th concentrations was carried out by resorting to external calibration with standards of Th prepared from single-element ICP-MS standard solutions (SPEX CertiPrep, Inc., Vernon Hills, IL, USA) for thorium. An analytical blank consisting of HAp–Th without polymers or DTPA was prepared in the same conditions. Bismuth was added to each sample at a concentration of 10 ppb to correct for sample matrix effects. Dose–response curves represent the percentage of Th recovered from the filtrate versus the chelate concentration (monomer). Results were expressed as the mean (±SD) from triplicates.

### 3.7. Kinetics of Th Extraction from the HAp–Th Subjected to the Chelates

Firstly, 5 mg of HAp–Th powder (9.3 ppm Th content as determined by ICP-MS) was added to a 1.5 mL solution of PEI-MC, PEI-MP, or DTPA at a concentration of 6.3 mM (monomer) in TBS buffer (50 mM Tris, 150 mM NaCl, pH 7.4). Each sample was prepared in triplicate. A centrifugation step (15,000 rpm, 15 min) was carried out at different times (2, 18, 42, 66, 90, 114, 162, 186, 210, 290, 354, and 504 h). Blank samples (absence of chelates) were also evaluated in the same conditions. Next, 500 µL of supernatant was recovered from two centrifugations (250 µL 2×) for each sample and digested in 5 mL of 67–70% HNO_3_ (Plasmapure plus degree, SCP Science) at 120 °C for 2 h. The digested samples were then evaporated to dryness at 90 °C using a heating block. Finally, 5 mL of 1.5% HNO_3_ was added to each tube, and samples were analyzed by ICP-MS (Perkin Elmer ELAN 9000) as previously described. Kinetic curves represented the percentage of Th recovered from the filtrate with a 6.3 mM chelate concentration (monomer) at the specified time. Results were expressed as the mean (±SD) from triplicates.

### 3.8. EXAFS of HAp–Th, PEI-MP–Th, and PEI-MP–Th–HAp

#### 3.8.1. Sample Preparation

Solid pellets were prepared by mixing HAp–Th (5mg) with polyethylene to obtain homogeneous solid pellets.

PEI-MP–Th was also prepared by using the same stock solution of Th(IV) as described above (Th(NO_3_)_4_ (0.1 M)). Then, 50 µL of Th(NO_3_)_4_), [Th] = 2.5 × 10^−3^ M pH = 1, was mixed with 250 µL of PEI-MP solution (5 mM of monomeric units) in Tris/NaCl buffer (50 mM, 150 mM). The pH was increased slowly to pH 7.0 by adding NaOH.

PEI-MP–Th–HAp was prepared by directly using the sample from the dose–response experiments of HAp–Th subjected to the chelates. Then, 1.5 mL of supernatant was recovered for each experiment. A centrifugation step (12,000× *g*, 5 min, 20 °C) was carried out on 10 kDa microcon^®^ centrifugal filter, allowing us to concentrate the sample for suitable EXAFS measurements.

#### 3.8.2. Data Recording and Processing

XAS data were recorded at the Th L_III_ edge (16,300 eV) on the MARS beamline at the SOLEIL synchrotron facility, which is dedicated to the study of radioactive materials. The optics of the beamline consisted of a water-cooled double-crystal monochromator for incident energy selection and horizontal focalization and two large water-cooled reflecting mirrors for high-energy rejection (harmonic part), vertical collimation, and focalization. All measurements were recorded in double-layered solution cells (200 µL) specifically designed for radioactive samples at room temperature. A 13-element Ge detector was used for data collection in the fluorescence mode.

Data treatment was carried out using ATHENA code of Demeter 0.9.26 package [31]. The E_o_ energy was identified at the maximum of the absorption edge. Background removal was performed using a pre-edge linear function. Atomic absorption was simulated with a cubic spline function.

#### 3.8.3. Data Fitting

The extracted EXAFS signal was fitted in R space without any additional filtering after Fourier transformation with a Hanning window in k^2^ and k^3^ using the ARTEMIS code of Demeter 0.9.26 package [31]. Phases and amplitudes were calculated with Feff7 code embedded in ARTEMIS code. Only one global amplitude factor S_02_ fixed to 1 and one energy threshold correction factor Δe_0_ were used for all paths. The agreement factor R (in %) and quality factor-reduced χ^2^ were both provided as an indication of the fit quality.

HAp–Th (Hanning window = 2.7–11 Å^−1^, fit range = 1–4 Å). The model used for phases and amplitude calculations was a Th_4_(PO_4_)_4_P_2_O_7_ crystallographic structure [26] where the coordination sphere of Th was composed of six monodentate and one bidentate phosphate. The first coordination sphere was adjusted with three contributions of oxygen atoms sharing the same Debye–Waller factor. The total number of oxygen atoms was set to nine atoms as the average for Th coordination. Two contributions of one and six phosphorus atoms were included in the fitting procedure. The triple Th–O–P and quadruple Th–O–P–O paths for the monodentate phosphorus contribution were also considered. The multiple scattering paths shared the same Debye–Waller and distance correction factors.

PEI-MP–Th and PEI-MP–Th–HAp (Hanning window = 2.7–10 Å^−1^, fit range = 1–4 Å). The model used for phases and amplitude calculations was described elsewhere [32]. The fitting procedure was the same as that used to fit previous EXAFS data of PEI-MP–Th (but synthesized differently, as described in Lahrouch et al. [17]). The first coordination sphere was fitted with a single scattering path of nine (fixed) oxygen atoms. A single scattering path of phosphorus atoms and the corresponding quadruple scattering path Th–O–P–O were also included in the fitting procedure. The addition of a single scattering path of chlorine atoms significantly improved the quality of the fit, as already observed elsewhere [17].

## 4. Conclusions

We showed herein, on the basis of strongly supported literature data and previous work we performed on actinide complexation with chelating polymers based on a PEI scaffold, that PEI-MP, i.e., the polyphosphonate analog of DTPA, can be considered as serious candidate for decontamination of Pu(IV) specifically targeted to the bones. Firstly, the PEI-MP, synthetized from a branched PEI (25 kDa), used without mass fractionation, did not show any toxicity toward bones constitutive cells, SAOS-2 and MLO-A5, unlike DTPA, which substantially decreased the viability at a concentration of 10 mM. Secondly, we demonstrated, using thermogravimetric analysis, that PEI-MP had a 15-fold higher affinity than DTPA toward hydroxyapatite, which constitutes the major part of the mineral bone matrix. We then successfully prepared hydroxyapatite contaminated with 0.7% Th(IV) (used as a Pu(IV) surrogate) and demonstrated through EXAFS experiments the full incorporation of this actinide into the bone mimicking matrix. Under the tested conditions, an optimum chelate concentration of 6.3 mM was sufficient to achieve maximum Th extraction with both compounds (DTPA and PEI-MP). However, the PEI-MP was able, in this case, to extract twice the amount of Th(IV), 29% versus 17%, than the gold standard DTPA. Additionally, when a kinetic study was performed over a 21 day period, this difference continued to increase, and the decontamination with the PEI-MP reached almost 50% at day 21, whereas it remained constant at 20% from day 1 with DTPA. Subsequent treatment with a fresh dose even increased the final level to almost 65%. In a more general context of actinide contamination, targeting the biological sites (lungs, liver, bones) that are involved in the sequestering of a substantial proportion of these radioelements could constitute a real advance in the field. This polymeric strategy, thus, provides a first, but nonetheless interesting complementary approach to the chelating therapy currently used based on Ca-/Zn-DTPA. Future studies aimed at tuning the kinetics and limiting the depletion of endogen cations are currently in progress.

## Figures and Tables

**Table 1:** EXAFS best-fit parameters for HAp–Th, PEI-MP–Th, and PEI-MP–HAp–Th under (pseudo)physiological conditions ^a^.

**Sample**	**First Coordination Sphere**	**Second Coordination Sphere**	**Fit Parameters**
HAp–Th	2.0 (1) O at 2.32 (3) Å σ^2^ = 0.0032 Å^2^	1 P at 3.21 (3) Å σ^2^ = 0.0074 Å^2^ 6 P at 3.82 (6) Å σ^2^ = 0.0181 Å^2^	S_02_ = 1.0 e_0_ = 1.86 eV R_factor_ = 3.3% Q = 15
	4.0 (1) O at 2.45 (2) Å σ^2^ = 0.0032 Å^2^		
	2.0 (1) O at 2.59 (5) Å σ^2^ = 0.0032 Å^2^		
PEI-MP–Th	9 O at 2.36 (1) Å σ^2^ = 0.0109 Å^2^	2.8 P at 3.88 (4) Å σ^2^ = 0.0927 Å^2^ 1.8 Cl at 3.13 (3) Å σ^2^ = 0.0117 Å^2^	S_02_ = 1.0 e_0_ = −0.13 eV R_factor_ = 5.4% Q = 88
PEI-MP–HAp–Th	9 O at 2.37 (2) Å σ^2^ = 0.0111 Å^2^	2.8 P at 3.82 (9) Å σ^2^ = 0.0171 Å^2^ *1.8* Cl at 3.14 (4) Å σ^2^ = 0.0096 Å^2^	S_02_ = 1.0 e_0_ = −0.34 eV R_factor_ = 5.5% Q = 52

^a^ σ^2^ is the Debye–Waller factor of the considered scattering path. S_02_ is the global amplitude factor, e_0_ is the energy threshold, R_factor_ is the agreement factor of the fit in percentage, and Q is the quality factor (reduced chi^2^) of the fit. Uncertainties given in brackets are related to the last digit. Numbers in italics were fixed. For HAp–Th, the sum of the coordination numbers of the oxygen atoms was fixed to 8, and the coordination numbers for the second sphere were fixed to the corresponding crystallographic phase of Th_4_(PO_4_)_4_P_2_O_7._ The coordination numbers for PEI-MP–Th were fixed to the values obtained in the previous study [17].

**Table 2:** Effect of cumulative dose administration.

	Th % Removal at d_12_	Total Th % Removal at d_21_
1 dose only from d_1_	38.2 ± 0.85	46.1 ^b^ ± 0.52
2 doses ^a^	35.8 ± 0.49	64.9 ^b,c^ ± 0.98

^a^ Independent experiment: medium was withdrawn at d_12_ and replaced with a fresh amount of 6.3 mM monomer PEI-MP. ^b^ Probability *p* (same mean as d_12_) < 0.005. ^c^ Probability *p* (same mean with one or two doses) < 0.005.

## Data Availability

The authors confirm that the data supporting the findings of this study are available within the article and its Supplementary Materials.

## Supplementary Materials

The following supporting information can be downloaded at: https://www.mdpi.com/article/10.3390/ijms23094732/s1.
