# Supplementary material for: Chelating Polymers for Targeted Decontamination of Actinides: Application of PEI-MP to Hydroxyapatite-Th(IV)
Source: Int J Mol Sci. 2022 Apr 25;23(9):4732. doi: 10.3390/ijms23094732 (PMC9100511; doi:10.3390/ijms23094732)
Supplement: Supplementary file 1 [file ijms-23-04732-s001.zip › ijms-1650037-supplementary.pdf]

Supplementary Materials :

2. Results and Discussion

2.5. Complementary Th Content in the Dose-Response and Kinetics Studies of HAp-Th Subjected to the Chelates

For each sample, the complementary part, i.e., 1 mL of the remaining supernatant plus the 5 mg of HAp-Th powder were mineralized as described in the manuscript (5 mL of 67–70% HNO_3, Plasmapure plus degree, SCP Science, at 120 ° C during 2 h). The digested samples were then evaporated to dryness at 90 °C using a heating block. Determination of the Th concentrations was carried out by resorting to external calibration with standards of Th prepared from single element ICP-MS standard solutions (SPEX CertiPrep, Inc.) for thorium. Results were expressed as mean (±SD) from triplicates.

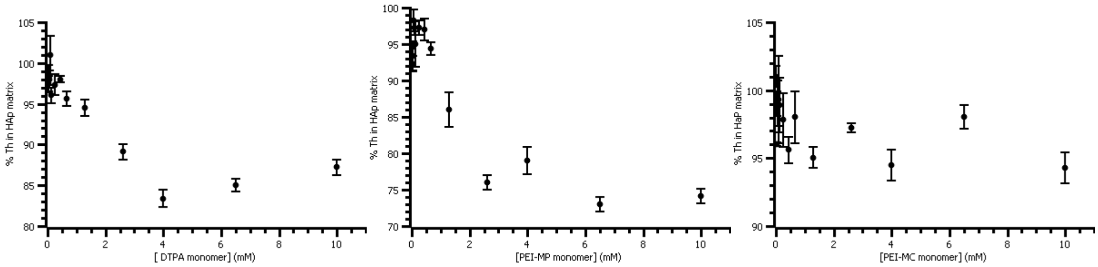

Figure S1. Complementary Th content (%) recovery for the dose-response experiments

2.7. Kinetics of HAp-Th chelate systems

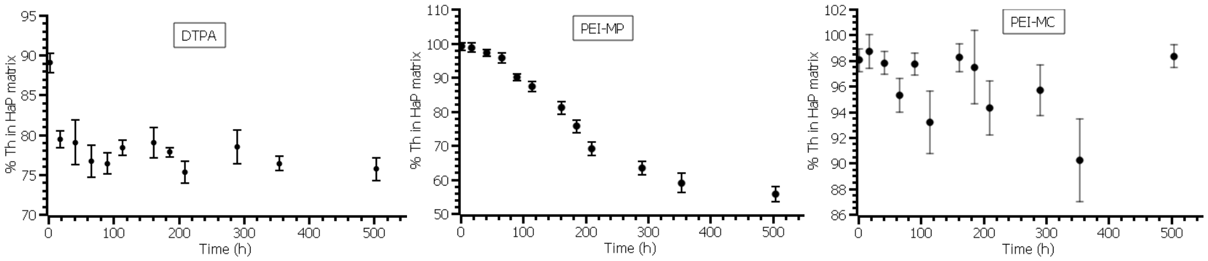

Figure S2. Complementary Th content (%) recovery for the kinetics experiments

Table S1. Complementary Th content recovery after a cumulative dose administration

	Th % recovery at d_{12}	Th % recovery at d_{21}
1 dose only from d_1		54.8 ± 2.92
2 doses [a]	64.3 ± 1.01	36.19 ± 0.89

[a] independent experiment: medium was withdrawn at d_{12} and replaced with a fresh 6.3 mM 1-mer PEI-MP

3. Materials and Methods

3.2. Synthesis of PEI Chelates

Counterions Titration into PEI-MC and PEI-MP

Sodium Titration (ICP-MS)

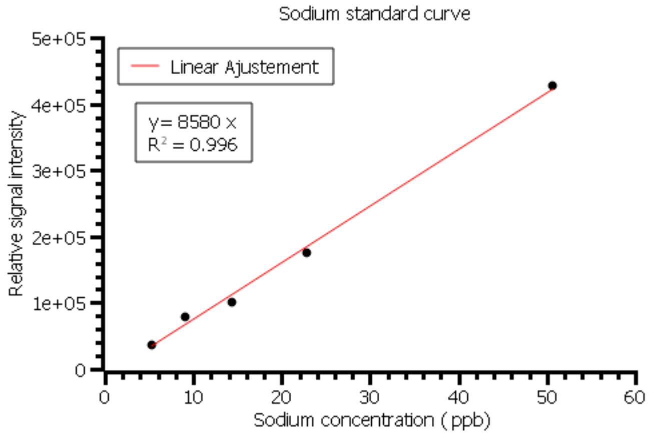

Figure S3. Na^+ titration curve

After mineralization of PEI-MC or PEI-MP (15 mg), 2.18 mg of sodium, 2.2 ± 0.2 Na / monomer was found for PEI-MC and 2.46 mg of sodium, 3.5 ±0.4 Na/monomer in the case of PEI-MP.

Chloride Titration (ion chromatography)

Chloride solution titration solutions were prepared from commercial standard solution (100 mg/L) as folow (results were expressed as mean from triplicates).

Table S2. Signal (peak high) versus chloride concentration

Signal (peak high)	[Cl^-] (mg/L)
0.51	3.91
1.17	7.81
2.56	15.62
5.24	31.25
12.01	62.5
27.2	125

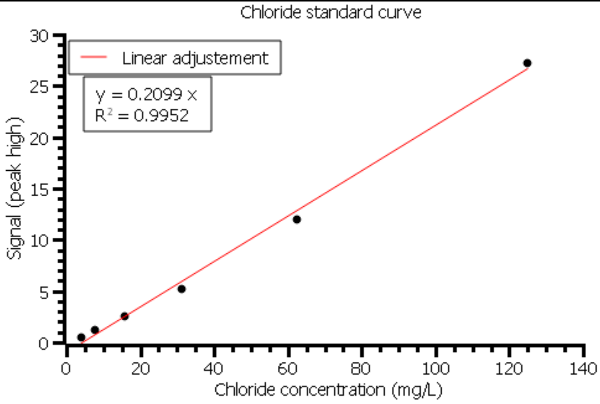

Figure S4. Cl^- titration curve

Table S3. Chloride counterion mass % content per polymer

Samples	Signal (peak high)	[Cl^-] (mg/L)	[PEI as monomer] (mg/L)	Cl^- (mass %)
PEI- MC (1/10)	0.85	4.05	3512	0.12
PEI-MP(1/10)	0.12	0.572	2320	0.02

The content of chloride anion was found to be negligeable for both PEI-MC and PEI-MP.

3.5. Contamination of HAp

Th Concentration at Each Washing Step of HAp

After contamination of HAp with $Th(CO_3)_4$, the HAp-Th powder was submitted to three cycles of centrifugation/washing steps with ultrapure water until no Th could be detected (ICP-MS) into the last filtrate. The first centrifugation step led to 3.40 ± 0.04 mg of Th. The 2nd filtrate contained 1.90 ± 0.06 mg of Th and no significative amount of Th was found in the last (third) filtrate.

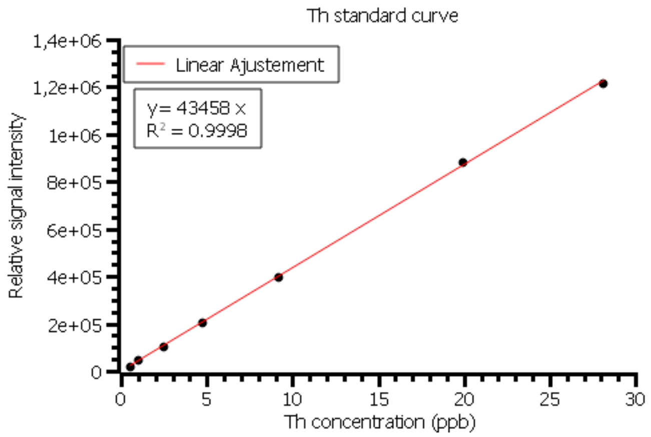

Figure S5. Th titration curve
